# Histological Evaluation of Single and Double-visit Direct Pulp Capping with Different Materials on Sound Human Premolars: A Randomized Controlled Clinical Trial 

**Published:** 2015-03-18

**Authors:** Sepideh Banava, Mahta Fazlyab, Haleh Heshmat, Faramarz Mojtahedzadeh, Pouria Motahhary

**Affiliations:** a *Department of Restorative Dentistry and Department of Dental Materials, Azad University, Dental Branch, Tehran, Iran; *; b *Iranian Center for Endodontic Research, Research Institute of Dental sciences, Dental School, Shahid Beheshti University of Medical Sciences, Tehran, Iran; *; c * Department of Restorative Dentistry, Azad University, Dental Branch, Tehran, Iran; *; d * Department of Orthodontics, Tehran University of Medical Sciences, Tehran, Iran; *; e * Department of Oral and Maxillofacial Pathology, Tehran University of Medical Sciences, Tehran, Iran*

**Keywords:** Calcium Hydroxide, Direct Pulp Capping, Mineral Trioxide Aggregate, MTA, Pulp Exposure, Vital Pulp Therapy

## Abstract

I**ntroduction: **The aim of this study was to evaluate the clinical and histological status of the pulp in sound human premolars after direct pulp capping (DPC) with four different DPC methods/materials. **Methods and Materials: **This study was conducted on eight volunteers who had to extract four first premolars due to orthodontic treatment. Subsequent to tooth isolation, standardized class I occlusal cavities were prepared and the buccal pulp horns were exposed. Then four different protocols of DPC were applied randomly: group A (control); calcium hydroxide lining paste (Dycal), group B; ProRoot MTA (standard double-visit method), group C; ProRoot MTA (single-visit method) and group D; calcium hydroxide injectable paste (Multi-Cal). The cavities were then restored and the patients were put on a six-week clinical follow-up and by the end of this period the teeth were extracted for histological evaluation. Data were analyzed with the Kruskal Wallis test and the level of significance was set at 0.05. **Results: **In terms of clinical symptoms and formation of hard tissue bridge (HTB), no significant differences were found between groups A, B and C (*P*>0.05); however, group D’s results were significantly different as they exhibited minimal HTB formation and excessive sensitivity (*P*<0.05). Inflammation was significantly lower in group B (*P*>0.05).** Conclusion: **Application of MTA during a single-visit protocol of DPC was clinically and histologically as successful as the standard double-visit method but the routine use of Multi-Cal as pulp capping material is questionable and should be reconsidered.

## Introduction

When an accidental/mechanical pulp exposure occurs, different vital pulp therapy (VPT) methods and more specifically direct pulp capping (DPC) is indicated [1, 2]. If proper considerations are adhered to, pulp self-protection by formation of a hard tissue barrier (HTB) beneath the capping material can be expected [3, 4]. Ideally if the treated pulp is healthy, the procedure is done in a bacteria free environment and the pulp is in contact with a biocompatible, bioregenerative and hard tissue inducing material, pulp preservation is likely provided that a bacterial tight seal guarantees the long-term outcomes [4-9].

Since the introduction of calcium hydroxide (CH) to dentistry by Codman [10], it has been the golden standard for DPC [10, 11]. CH has antibacterial properties due to its high alkalinity (pH value of 11-12) in natural powder state [10, 11]. Contemporary cavity liner formulations such as Dycal (Dentsply, Tulsa, OK, USA), coming in the form of paste self-curing products, are less caustic and may not show pH values more than ~10 depending on their buffering additives [1, 12]. It is shown that CH stimulates the formation of HTB [10-12]. However, microleakage under the tooth restoration due to the solubility of CH and presence of tunnel defects in the HTB caused by tissue degradation property of this material, imply a negative impact on treatment outcomes [2, 10, 11]. 

In an attempt to overcome this deficiency, other DPC agents have been introduced. Pulpdent Multi-Cal (Pulpdent Corporation, Watertown, MA,USA) is an injectable premixed CH containing paste packed in syringes that offers the advantage of easy placement of the material in the cavity floor [13]. The material bulk should be covered with a base protective material and the manufacturer offers a resin-based, light-cured base named Lime-Lite (Pulpdent Corporation, Watertown, MA, USA) [14]. 

One study reported the mean 12-month results of pulpotomy in permanent incisors with Multi-Cal; all of the treated teeth showed radiographic evidence of HTB formation within 1 to 3 months after pulpotomy [15]. On the other hand, in a study on human permanent teeth, four-month histological response after partial pulpotomy with either Dycal or Multi-Cal revealed that while all pulps treated with Dycal showed tissue healing and HTB formation, there were notable numbers of necrotic pulps and bacterial penetration in samples treated with Multi-Cal [16]. A systematic review over the formation of HTB in human teeth after DPC, failed to perform a meta-analysis due to heterogenic results that were found between the studies; however, the review stated that most of the evaluated investigations were suggestive of the HTB formation after DPC with different components of CH [17].

As a bioactive material, mineral trioxide aggregate (MTA) has been subject of several investigations which started as simple laboratory tests and nowadays have involved human subjects. MTA is biocompatible and presents good antimicrobial effects [18, 19], provides excellent sealing [20] and is capable of stimulating pulp and periapical regeneration [11, 21, 22]. MTA is being commonly used for different types of VPT but has some drawbacks including handling difficulties, long setting time and tooth discoloration [19, 23-25]. 

Many attempts have been done to overcome these disadvantages. In this regard it is stated that application of light-cured glass ionomer on partially set MTA after 45 min does not interfere with the setting of either materials [25] and this can ensure a single-visit DPC treatment without the need for re-opening the cavity to allow for complete setting of MTA, as suggested by the manufacturer [26]. Compared to a 24-h waiting time, a 45-min delay seems more favorable but it still seems too long for clinical application.

Many studies considering the effects of different biomaterials on dental pulp, have used DPC [19, 21, 22, 27, 28]. The aim of this randomized single-blind clinical trial was to evaluate the clinical and histological responses of pulp in sound human premolars after application of four DPC methods (*i.e.* Dycal, Multi-Cal, MTA as the standard double-visit method, and MTA as a single-visit method) during a 6-week period. The null hypothesis was that all tested methods/materials can reveal similar results and thus can be interchangeably used in clinical practice.

## Materials and Methods

The protocol for this study was approved by Human Ethics Committee of Azad University, Dental Branch, Tehran, Iran. The project was registered online (ClinicalTrials.gov Identifier NCT01468480). After performing a pilot study on four samples, using 2 proportions submenu from sample size calculation menu of Minitab, the minimum estimated sample size for each group was estimated to be eight.

Participants were chosen according to the following inclusion/exclusion criteria: periapical radiographs without any periradicular radiolucency, no sensitivity to percussion/palpation and biting, no sensitivity after application of cold test with Green Endo Ice (Colten, Whaledent, NJ, USA) for 15 sec and no sensitivity to heat after applying tempered gutta-percha. Each tooth was compared to its adjacent sound tooth as control [6, 29].

**Figure 1:**
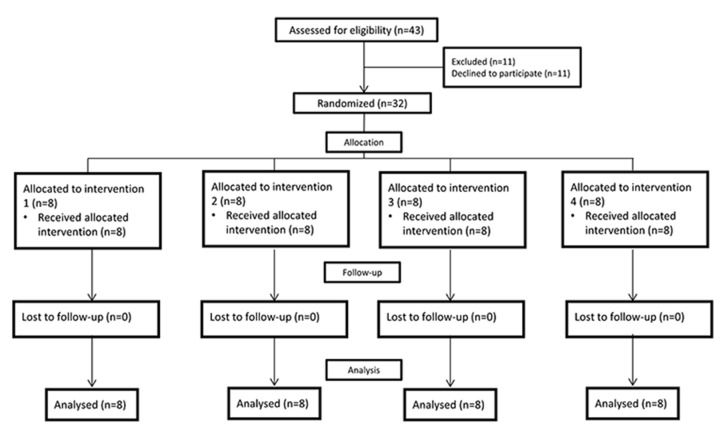
Process of enrolment, randomization, allocation, intervention and follow-up

Twelve patients with mean age of 16.5 (ranging from 13 to 20 years old) were found eligible, each one having four intact and fully erupted first premolars that were scheduled to be extracted for orthodontic treatment. Four patients refused to participate in the study. All the remaining eight participants (and their parents) agreed to the conditions of the study and signed the consent form (Figure 1). 

Before operation, a complete medical history was taken to ensure the absence of any systemic disease and sensitivity to local anesthesia or dental materials. Then patients were requested to rinse their mouth with 0.2% chlorhexidine gluconate (Behsa Co, Tehran, Iran). Following infiltration of 2% lidocaine containing 1:80000 epinephrine (Darupakhsh, Tehran, Iran) and placement of rubber dam, a Class I cavity was prepared with a sterile as-received 008 diamond fissure bur (Komet, Brasseler GmbH & Co., Lemgo, Germany) installed on a high speed handpiece under copious water irrigation [30, 31]. All the cavities had depths similar to the bur length (~3 mm). Then the buccal pulp horn of each tooth was exposed through the cavity floor with a 0.5 mm-diameter round diamond bur 835 (Komet, Brasseler GmbH & Co., Lemgo, Germany). The exposed area was rinsed with sterile saline solution and hemostasis was completed with sterile cotton pellets saturated with sterile saline which was kept in place for 10-20 sec. 

For each patient four first premolars were prepared in turn and then the exposed pulps were randomly capped according to a computer generated randomization pattern (toss of coin) from www.random.org, as follows: 

***Group A.*** self-cured calcium hydroxide lining paste (Dycal, Dentsply, Tulsa, OK, USA) was mixed and applied to the exposure site. After setting, it was covered with a 1-mm thick layer of light-cured resin modified glass ionomer (RMG) (Fuji II LC, GC, Japan).

***Group B. ***ProRoot MTA (Tooth-colored formula, Dentsply, Tulsa Dental, Tulsa, OK, USA) was prepared according to the manufacturer’s instructions and applied over the exposure site.

A moistened sterile cotton pellet was placed over the material and the cavity was temporarily restored (Coltozol, Asia Chemi Teb. Co., Tehran, Iran). The permanent restoration was placed after 24 h [26]. 

***Group C;*** ProRoot MTA was prepared and applied similar to group B. Then a wet sterile cotton pellet was placed on the MTA bulk for 15 min [32], and afterwards the material was covered with a 1-mm thick layer of RMG, the same as group A. The tooth was permanently restored on the very same appointment. 

***Group D;*** In this group, Pulpdent Multi-Cal liner (Pulpdent Corporation, Watertown, MA,USA) was placed over the exposure area and then it was covered with a resin-based base material (Lime-Lite, Pulpdent Corporation, Watertown, MA,USA) which was light cured for 40 sec. 

For all samples the restoration of the cavity was completed in the same session except for the teeth in group B that were restored after 24 h. All of the cavities were restored with a self-etch one-step adhesive system (G-Bond, GC, Japan) and composite resin (A2, Gradia Direct, GC, Japan). All the procedures were done by one calibrated operator. 

***Follow-up***

The patients were fully justified about the probable signs/symptoms (such as spontaneous pain, swelling, sensitivity to cold/heat/percussion/chewing and, *etc*.) during the six-week follow-up period. The patients were contacted weekly and all their clinical symptoms were meticulously recorded. Any spontaneous or prolonged pain was interpreted as failure. In case of treatment failure or patients’ change of mind, they were excluded immediately and their teeth were extracted and sent for histological evaluation.

***Tooth extraction and histological assessment***

By the end of sixth week, after doing pulp sensitivity tests and taking periapical radiographs by the main operator, the teeth were extracted. The roots were sectioned in the mid-root area, fixed in 10% buffered formalin, and then demineralized in 10% formic acid. The samples were embedded in paraffin and a total number of 5 to 10 axial sections with 5 μm thicknesses were prepared and stained with Hematoxylin and Eosin (H&E) [30]. In this study, the process of tooth extraction and preparation for histological assessment followed the suggested protocol by International Standard Organization, ISO 7405 [30, 33].

According to this standard, specimens were histologically assessed in terms of the appearance and quality of the HTB, the organization of pulp cells mainly in odontoblastic layer adjacent to the exposure area, and inflammatory status of the underlying pulp [30, 33] using an optical light microscope with magnification of 4×, 20×, 40× and 100× (Nikon, Plan Flour, Japan) by an oral pathologist who was blind to the procedure. The inflammatory infiltrate was scored as follows: *score 0*- no inflammation, *score* *1*-mild inflammation, *score* *2*-moderate inflammation, *score* *3*-severe inflammation and *score* *4*-abscess formation or extended lesions not localized to the tissue beneath the material. Moreover, the degree of bridging over the capped area of the pulp was scored as *0*-HTB not formed, and *1*-HTB formed. The appearance/quality of HTB was classified as: *0*-resembling natural dentin, *1*-atubular dentine and *2*-presence of tunnel defects. The clinical sensitivity was scored as *score* *0*-Non-sensitive and *score* *1*-sensitive. Due to ethical reasons the negative control group (exposed pulps without applying a pulp capping material) was omitted. Statistical analysis was done with the Kruskal Wallis test and *P-*values less than 0.05 were considered statistically significant.

## Results

All 12 patients including 4 male and 8 female volunteers were available for the final evaluation (Figure 1). None of the patients had complained of any spontaneous lingering pain during the follow-up duration. All teeth were extracted atraumatically.

***Clinical signs/symptoms***

None of the teeth in groups A, B and C had clinical symptoms (*score 0*). Sensitivity to heat was reported in two teeth in group D, but the difference among the groups was not statistically significant (*P*>0.05). Also five patients in this group had sensitivity to cold (*score 1*), which was significantly different from the other groups (*P*<0.05) (Table 1). 

***Formation of dentinal bridge***

Table 2 represents the formation of HTB in the study groups. In group A (Dycal), seven specimens showed HTB (*score 1*), while one case showed no evidence of bridging. In groups B (MTA; double-visit) and C (MTA; single-visit), HTB was evident in six samples and in group D (Multi-Cal), only two samples had formed the HTB. There was no significant difference among groups A, B and C in terms of HTB formation, while the amount for group D was significantly lower (*P*<0.05). 

***Appearance of HTB***

**Figure 2:**
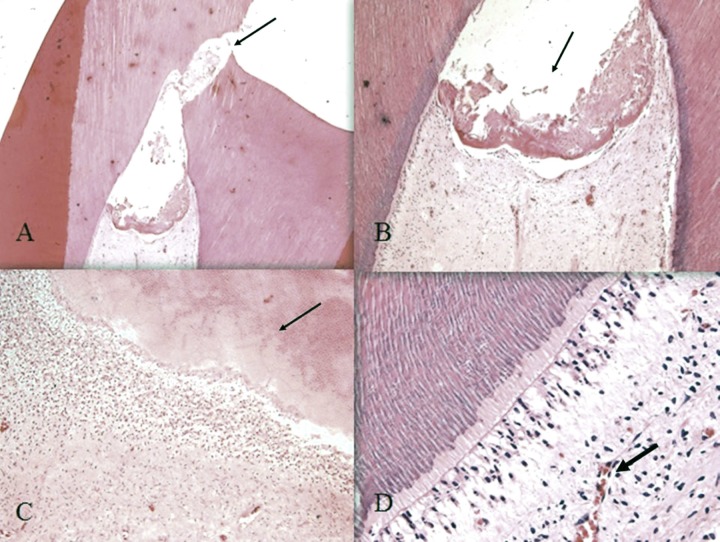
Histological presentation of hard tissue bridge (HTB) beneath White ProRoot MTA in group B: *A)* Exposure site (arrow) (10× magnification); *B)* formation of HTB in exposure site (arrow) (40× magnification), *C)* Atubular dentin (arrow) (100× magnification) and *D)* lymphocyte infiltration (arrow) in the underlying pulp (200× magnification)

In all groups, the formed HTB showed an atubular appearance and the Kruskal-Wallis test did not show any significant differences among the groups (*P*>0.05). Figure 2 shows the histological appearance of the HTB beneath MTA in one of the samples in group B.

***Inflammatory response***

Generally typical infiltration of inflammatory cells, especially macrophages and lymphocytes, was observed. Table 3 represents the inflammatory cell response scored on a scale from 0 to 4. The results indicated no significant difference among groups A, C and D. However, in group B the inflammatory response was significantly lower (*P*<0.05). None of the specimens showed necrosis/abscess (*score 4*). 

## Discussion

This was a single-blind randomized controlled clinical trial on 32 sound human premolars divided into four experimental groups that were pulp capped with Dycal, ProRoot MTA (double-visit), ProRoot MTA (single-visit) and Multi-Cal as four DPC methods. The histological response in terms of HTB formation and inflammatory infiltration, revealed similar responses in all experimental groups except for Multi-Cal that had the least and most amount of HTB and inflammation, respectively. The results also proved the hypothesis regarding the similarity of results after DPC with MTA in single or multiple sessions. 

A very important aspect of this study is the use of sound teeth. The classic study by Kakehashi *et al*. [34], established the role of bacteria in pulp health and necrosis. In a germ-free environment, the pulp demonstrated the ability to heal and deposit additional dentin material. In the presence of bacteria, pulpal demise was inevitable. This fundamental premise is integral to the success of all vital pulp procedures. Many research studies on evaluation of the effects of different materials on pulp, have been conducted on human or experimental animal sound teeth [18, 21, 27, 28]. The present study was conducted on caries free human teeth, in order to be able to justify the probable treatment failures by the material used. There is no doubt that the results of this study do not reveal the true effects of used biomaterials in more close to real circumstances when the pulp is already inflamed. It is highly recommended to conduct more studies on cariously exposed teeth and longer follow-up duration.

**Table1:** Clinical sensitivity [N (%)] to cold after pulp capping with different biomaterials and techniques

	**0-Non-sensitive**	**1-Sensitive **	**Total**
**Dycal**	8 (100)	0 (0)	8 (100)
**MTA double-visit**	8 (100)	0 (0)	8 (100)
**MTA single-visit**	8 (100)	0 (0)	8 (100)
**Multi-Cal**	3 (37.5)	5 (62.5)	8 (100)

**Table 2:** Deposition of the hard tissue barrier (HTB) [N (%)] after pulp capping with different biomaterials and techniques

	**0-Non-sensitive**	**1-Sensitive **
**Dycal**	1 (12.5)	7 (87.5)
**MTA double-visit**	2 (25)	6 (75)
**MTA single-visit**	2 (25)	6 (75)
**Multi-Cal**	6 (75)	2 (25)

**Table 3:** Scoring the inflammatory response of the pulp [N (%)] after pulp capping with different biomaterials and techniques

	**0-None**	**1-Mild**	**2-Moderate**	**3-Severe**	**4-Abscess**	**Total**
**Dycal**	1 (12.5)	2 (25)	4 (50)	1 (12.5)	0 (0)	8 (100)
**MTA double-visit**	3 (37.5)	4 (50)	0 (0)	1 (12.5)	0 (0)	8 (100)
**MTA single-visit**	0 (0)	3 (37.5)	3 (37.5)	2 (25)	0 (0)	8 (100)
**Multi-Cal**	0 (0)	3 (37.5)	3 (37.5)	2 (25)	0 (0)	8 (100)

Similar to all other methods of VPT, DPC has a high success rate if the following conditions are met: *i*) the uninflamed pulp tissue is covered; *ii*) hemorrhage is properly controlled; *iii*) a non-toxic and bioregenerative capping material is applied and *iv*) the capping material and future restoration provide hermetic seal [35-37]. It is shown that after DPC the formed HTB is porous and this feature reduces as the bridge gets older and thicker, but never fades away [38]. In other words, HTBs are not true protective barriers for the underlying pulp. Thus double sealing of the cavity is suggested so that the HTB would act as a secondary barrier to protect the pulp, no matter how porous it might be [1, 6]. In the current study complete sealing of the cavity was performed with glass ionomer. This porous feature *aka.* “tunnel defects” indicates severe pulp injury at the time of intentional exposure [22, 38, 39] or bacterial contamination [22]; therefore preventing the pulp injury, by means of copious water cooling during cavity preparation, tooth isolation and use of as received sterilized burs for each patient, were the important aspects of the present study. 

 HTB in all groups showed an atubular appearance. The number of teeth with HTB formation was the highest in Dycal group, followed by MTA in double- and single-visit methods. However, the tunnel defects were notable in teeth treated with Dycal. The findings released by Iwamoto *et al.* [1] indicated that CH and MTA were similar in inducing HTB, but the 3-month results of the study by Leye *et al.* [12], showed higher amounts of HTB with MTA compared to CH. The current study showed similar results between CH and MTA groups. The difference could be due to the duration of follow-up.

Although an SEM study evaluating HTB following pulp capping with MTA reported the formation of neodentinal bridge after 2 weeks [22], we chose a six-week period of follow-up, because one month is the shortest time needed for histological detection of HTB, and this delay can reflect the time required for proliferation, migration, and differentiation of new replacement secondary odontoblasts and formation of HTB [40].

As a bioactive material, MTA has been subject of several investigations which started as simple laboratory tests and nowadays have involved human subjects, showing that MTA is biocompatible and presents good antimicrobial effects [18, 19], provides excellent sealing [20] and is capable of stimulating pulp and periapical regeneration [11, 21, 22]. The prolonged setting time of MTA happens to be a clinical weakness that may prevent its routine use as a DPC agent in single visit restoration of deeply carious teeth [5, 10]. Clinical and histological results of this study showed that the single-visit method with MTA, along with immediate restoration resulted in an acceptable degree of success. As stated by Ballal *et al.* [41], placement of glass ionomer over partially set MTA did not affect its setting and glass ionomer setting was not affected by the presence of MTA, either. In this study in teeth treated with MTA single-visit method, a sterile saline wet cotton pellet was placed over the MTA to provide primary setting hydration and the cotton pellet was removed prior to continuing with restoration [32]. Similarity of treatment outcomes to those teeth treated with a double-visit standard method confirms this clinical application. The bridges formed in both MTA groups were thicker and more integrated compared to the other groups. It could be the result of MTA biocompatibility and its profound sealing [1, 5, 6, 10, 29]. On the other hand the application of glass ionomer cement as a base with physicochemical adhesion over the CH and MTA resulted in better sealing of the exposure site compared with the application of a resin-based material over the Multi-Cal that resulted in fewer bridge formation. Six out of eight teeth in Multi-Cal group showed no HTB formation, which might lead to the assumption of a lower sealing ability of this resin-based material in deep parts of the cavity, as stated by Subay *et al.* [16].

Another important issue in the current study was the inflammatory reaction to the procedure and materials. A total of 28 specimens showed different grades of inflammation, while only 4 were totally non-inflamed (*score-0*). Similar to the study by Scarano *et al.* [29], the present results confirms that inflammation of the pulp is reversible and does not provoke pulp necrosis. Teeth capped with MTA (single- or double-visit method) showed lower inflammation compared to the other groups. All specimens with inflammation showed chronic infiltrates. 

The last but not the least is the poor relation between clinical symptoms and the histological condition of the pulp which is supported by the results of the current study for the first three groups. Also it is noteworthy that clinical findings of the teeth treated with Multi-Cal indicated five patients with sensitivity to cold while histologically they had no HTB formation.

## Conclusions

The use of MTA during single-visit DPC was as successful as Dycal and standard double-visit application of MTA. Application of Multi-Cal resulted in poor clinical and histological findings, and therefore may not be the material of choice for DPC. 
